# Are you always working in the dark? The impact of limited daylight exposure on radiologists’ health

**DOI:** 10.1186/s13244-026-02248-x

**Published:** 2026-04-01

**Authors:** Anna Landsmann, Cassandra Rovetto, Fabienne Knöpfli, Rahel A. Kubik-Huch

**Affiliations:** Department of Radiology, Kantonsspital Baden, Affiliated Hospital for Research and Teaching of the Faculty of Medicine of the University of Zurich, Baden, Switzerland

**Keywords:** Radiology department (Hospital), Workplace environment, Lighting, Occupational health

## Abstract

**Abstract:**

Radiologists spend most of their day in dimly lit reading rooms, for controlled lighting is essential for accurate image interpretation. However, prolonged exposure to such environments poses health risks, including burnout, depression, and circadian disruption. These risks not only affect individual well-being, but also contribute to workforce shortages; radiology’s association with dark and isolating workplaces hampers recruiting young talent in radiology at a time when there is a shortage of skilled workers. Natural light, crucial for vitamin D synthesis, serotonin regulation, and sleep–wake cycles, is often missing in radiology departments. European workplace regulations emphasize daylight access, but exemptions for radiology highlight a gap between occupational health needs and imaging requirements. This article explores the health implications of limited daylight exposure, focusing on radiologists, and compares legal frameworks in Germany, Switzerland, and the European Union.

**Critical relevance statement:**

Working in dark environments not only threatens radiologists’ health, but also negatively impacts performance and professional image and is often overlooked in architectural planning and daylight regulations. This article highlights these issues through an analysis of current studies and legal frameworks.

**Key Points:**

- Poor lighting environments contribute to burnout and diagnostic fatigue in radiologists.
- Radiology reading rooms often remain exceptions to workplace lighting regulations.
- Integrating daylight and ergonomic lighting enhances radiologists’ health.

**Graphical Abstract:**

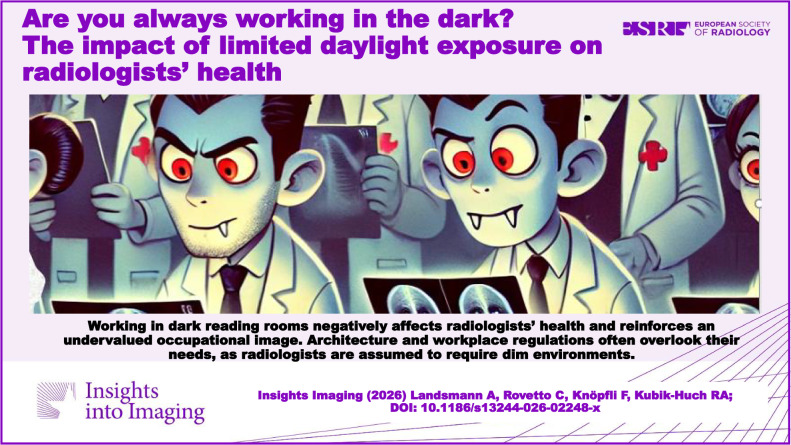

## Introduction

“Are you always working in the dark?” is a frequent question asked by colleagues from other medical disciplines or by patients when entering the reading room. While a dark environment is necessary for optimal image quality and reporting, adequate light exposure is essential for maintaining a healthy work environment.

Radiologists spend most of their working hours in dimly lit reading rooms to ensure optimal image quality when reading diagnostic images. The low light levels help prevent glare and improve contrast, allowing for accurate interpretations. While these dark environments are essential for precise diagnoses, the lack of natural light can negatively affect the health and well-being, increasing the risk of burnout and depression in radiologists [1].

Daylight exposure stimulates the production of vitamin D, which is important for immune function and metabolism [2]. Low vitamin D levels have been associated with mood disorders; however, causality remains debated, and evidence from occupational settings, such as radiologists are limited [3, 4]. Additionally, natural light triggers the release of serotonin, a neurotransmitter that regulates mood, enhances focus, and supports mental clarity [5, 6]. Insufficient exposure to daylight can disrupt this process, leading to conditions such as depression and anxiety. The circadian rhythm, which controls the sleep–wake cycle, is also influenced by daylight, helping to regulate sleep patterns. Lack of exposure to natural light can lead to sleep disturbances and fatigue [7, 8]. Therefore, adequate daylight exposure is crucial for maintaining mental health, energy levels, and overall productivity.

Other professional groups, as well as patients and the public, often perceive radiologists as “lone wolves in their dark caves”. This stereotype contributes to reduced interest in radiology among medical students, further aggravating workforce shortages [9] despite the increasing number of examinations in the past years [10].

Various regulations emphasize the importance of natural light in the workplace to ensure good health and well-being for employees [11–13]. These legal frameworks reflect the growing recognition of the role of natural light in regulating mood and overall health, encouraging its integration in the workplace. The job image, however, has led construction planners to often place radiology departments in darkened rooms, as radiologists do not want or need daylight.

This article focuses on the negative impact of limited daylight exposure in reading rooms on radiologists’ health and compares the current laws in Germany, Switzerland, and the European Union addressing light exposure at work.

## Materials and methods

This article is based on a comprehensive PubMed literature search of original research and reviews published between 2000 and 2025. Key search terms included “light deprivation AND circadian rhythm”, “light deprivation AND vitamin D”, “vitamin D deficiency AND depression”, and “radiologists AND burnout” (yielding ~2000 initial, often overlapping hits). Articles were screened by title and abstract for relevance (excluding non-English publications), with > 100 full-text articles reviewed before final inclusion. Legal frameworks and workplace regulations were analyzed using official legislative texts from Germany, Switzerland, and the European Union concerning light exposure and workplace design in radiology.

## The effect of darkness on psychic and physical health

Natural daylight is a key regulator of brain metabolism, cognitive function, mood, and physiological homeostasis. The circadian system, controlled by the suprachiasmatic nucleus, synchronizes the sleep–wake cycle with external light [14]. Daylight suppresses melatonin production, promoting alertness and cognitive performance, while darkness promotes melatonin release to regulate sleep. Chronic exposure to dim environments, as in radiology reading rooms, disrupts this balance, contributing to sleep disturbances [14, 15]. Light also modulates the hypothalamic–pituitary–adrenal axis, influencing cortisol secretion and stress response [16], which, combined with poor sleep, is particularly prone to cause chronic fatigue and affect diagnostic performance [17, 18].

In addition to these direct neurobiological effects, sunlight is essential for vitamin D synthesis, which supports bone metabolism, immune defense, and neurotransmitter regulation. Vitamin D receptors in the hippocampus and amygdala link it to emotion and cognition. Deficiency may impair serotonin and dopamine pathways, increasing vulnerability to depressive symptoms [5, 19]. While the causal relationship remains debated [20, 21], observational studies associate low vitamin D levels with mood disorders [22, 23]. Healthcare workers with limited sunshine exposure, such as radiologists, may be more likely to have lower vitamin D levels and higher rates of depressive symptoms [24]. Public Health Guideline 56 from the National Institute for Health and Care Excellence defines low vitamin D status as a plasma concentration < 25 nmol/L. It recommends daily supplementation (safe intake of 400 IU/day) for people with limited sunlight exposure to mitigate deficiency-related health risk [25].

Interventions, including bright-light therapy [26] and optimized workplace lighting [27], have demonstrated improvements in mood, cognition, and overall well-being, emphasizing the need for tailored lighting strategies in radiology to mitigate the long-term effects of working in dark environments.

## Legal frameworks on light exposure in Europe

Most European workplace regulations recognize the health benefits of adequate lighting, yet radiology reading rooms are often treated as exceptions due to diagnostic requirements (Table 1). Radiology reading rooms typically maintain low illumination levels of 20–40 lux, with monitor brightness ≥ 350 cd/m^2^ (Candela per square meter). Subspecialties, such as mammography, often require even lower levels (≤ 20 lux) and higher monitor brightness (≥ 420 cd/m^2^) to ensure optimal contrast [28, 29]. In other specialties where light is crucial for diagnostic accuracy—e.g., in examination rooms (500 lux) or operating rooms (≥ 3000 lux)—illumination levels are 10–100 times brighter than the radiology environment [30].

**Table 1:** Legal frameworks on light exposure in Germany, Switzerland, and the European Union

Jurisdication	Germany	Switzerland	European Union
Mandatory daylight access	Arbeitsstättenverordnung (ArbStättV) mandates natural light whenever possible, supplemented by artificial lighting when inadequate.	Arbeitsgesetz (ArGV 3/4), Chapters 3 and 4 emphasize natural light for circadian stability; permanent workplaces (> 2.5 days/week) require windows or counteracting measures (mandatory for new buildings); natural light is recommended in break areas.	Directive 89/654/EEC requires workplaces to receive sufficient natural light “as far as possible.”
Special provisions for radiology reading rooms	Explicit exemption: Radiology departments/reading rooms are exempt due to the need for controlled low-light conditions for image interpretation.	No explicit radiology exemption mentioned; general requirements apply, but diagnostic needs may allow practical adaptations via countermeasures.	No specific radiology exemption; remains a gray area with implementation variability across member states.
Recommendations	DIN 6868-157 classifies reading rooms as room class 1, limiting illumination to < 50 lux and defining permissible veiling luminance.	Article 15 addresses glare prevention; the SUVA guidelines support daylight compensation and ergonomic lighting to ensure health protection.	Focuses on general adequacy of lighting (natural and artificial); recognizes light’s impact on melatonin (mainly in shift work context); no prescriptive radiology-specific rules, leading to inconsistent enforcement among member states.

In Germany, the *Arbeitsstättenverordnung (ArbStättV)* mandates natural light whenever possible, supplemented by artificial lighting when natural light is inadequate. Radiology departments are exempt, since controlled low-light conditions are needed for image interpretation [11]. *DIN 6868-157* further specifies that radiology reading rooms fall into room class 1, limiting illumination to < 50 lux and defining permissible veil luminance [31].

In Switzerland, Chapters 3 and 4 of the *Arbeitsgesetz (ArGV 3/4)* emphasize natural light as essential for circadian stability, recommending natural light in break areas [32]. Article 15 of Chapter 3 addresses the phenomenon of glare, which can impair vision. It also requires permanent workplaces (> 2.5 days/week) to have windows or to provide counteracting measures to ensure overall health protection, being mandatory for all new buildings in Switzerland [33].

In the European Union, *Directive 89/654/EEC* states that “workplaces shall, as far as possible, receive sufficient natural light,” though implementation varies among member states, and radiology remains a gray area in lightning policies. They also recognize light’s impact on melatonin production in the context of shift work [34]. This Directive’s broad minimum requirements allow significant implementation variability and inconsistent enforcement across member states, functioning more as a framework than prescriptive rules for specialized settings like radiology. This patchwork approach highlights the need for stronger EU-level guidance to standardize enforcement and better balance diagnostic accuracy with occupational health in radiology.

The shift from film to digital imaging has reshaped these standards. In the era of film technology, the brightness of light boxes was the only relevant factor. In Germany, the standard of the *Deutsches Institut für Normung* (DIN) for approving light boxes has been replaced by the above-described DIN standard, which focuses on the luminance, contrast, and calibration of monitors. Device approval now considers the actual room lighting [31]. In Switzerland, the *Schweizer Unfallversicherungsanstalt* (SUVA) guidelines provide a checklist with a focus on daylight compensation and ergonomic lighting [35], while the European standard EN 12464-1 [13] has been updated to address digital diagnostics in terms of glare and reflection.

Despite these frameworks, radiologists still face prolonged darkness, exceeding 8–10 working hours, with limited daylight exposure. Evidence on its psychological impact has triggered calls for revised standards that better balance diagnostic accuracy with occupational well-being [28].

### Radiologists at risk for depression and burnout

Radiologists, among other physicians, are increasingly recognized as being at high risk for burnout and depression due to the unique demands of their profession [1]. Burnout not only undermines radiologists’ well-being but also affects diagnostic accuracy and patient outcomes. It is characterized by emotional exhaustion, depersonalization, and reduced personal accomplishment, all of which correlate with impaired performance and a higher risk of depression. Key contributing factors include chronic light deprivation, which disrupts circadian rhythms and induces fatigue and mood instability; high workload and diagnostic pressure, which increase cognitive strain; and limited patient interaction, which fosters professional isolation and emotional exhaustion [36].

A German study reported burnout in 76.7% of radiologists, strongly associated with an excessive workload, poor sleep, poor working conditions, and low job satisfaction [37]. Similar rates were reported in subspecialties: 80.5% of musculoskeletal radiologists and 78.4% of breast radiologists experienced at least one burnout dimension, mainly emotional exhaustion and depersonalization [38, 39]. Italian radiologists with effort-reward imbalance or overcommitment exhibited significantly higher depressive and anxiety symptoms [40]. Risk factors include heavy workload, long hours in dimly lit environments, insufficient recovery time, and poor work–life balance [41].

Taken together, these findings underline that burnout and depression are widespread among radiologists and driven by both organizational and environmental stressors. Targeted workplace and policy interventions are urgently needed to safeguard their mental health and maintain diagnostic quality.

## Discussion

Despite the well-established benefits of daylight for mental health, the influence of workplace design on radiologists is frequently overlooked, even amid substantial investments in hospital redesigns [42]. Larsen et al and Argawal et al advocate for comprehensive radiology workspace redesigns to enhance both cognitive performance and well-being, proposing differentiated work zones, dedicated reporting areas, and optimized lighting to reduce occupational stress and improve productivity [27, 43].

Earlier research by Brennan et al and Pollard et al demonstrated that ambient lighting could affect the interpretation of wrist and chest X-rays. Although digital radiography has mitigated some of these limitations, ambient light remains a relevant factor for optimal diagnostic accuracy, particularly in nuanced or low-contrast imaging [29, 44]. High contrast remains essential for accurate medical image interpretation. Ambient light reduces contrast through reflections and glare and disrupts ocular adaptation to stable luminance levels. Large differences between screen brightness and ambient light force constant readjustment, causing visual strain and fatigue, particularly when glare is present. Conversely, excessively dark environments also impair visual performance. Optimal conditions are achieved with dim, indirect lighting behind the screen, minimizing glare while supporting visual comfort and diagnostic accuracy [28].

Radiology reading rooms often feature blue-enriched ambient light to match the wavelength spectrum of diagnostic monitors. However, blue light exposure—primarily from monitors—carries a dual role: while it can reduce glare, prolonged exposure may disrupt circadian rhythm [7] and contribute to computer vision syndrome—a pattern of symptoms of eyestrain, headaches, blurred vision, and dry eyes. Blue-light filtering lenses can mitigate eye fatigue [45], though they may feel uncomfortable for non-glasses wearers. Wentzel et al found that actual blue light radiance in radiology settings remains approximately 10,000 times below recommended safety thresholds [46].

The optimal setup for a radiology reading room can be achieved by installing adjustable light-emitting diode (LED) fixtures or wall-mounted soft-glow lights. These provide subtle brightness during breaks and non-reading tasks and can be dimmed during image review. In contrast, even dimmable veiling should be avoided, as it increases glare through reflections on the monitor. Architectural redesigns can also incorporate dedicated zones: low-light areas for image interpretation and brighter daylight areas for breaks, a concept which is described as the Eudaimonia Machine by the architect David Dewane and applied to radiology workplaces by Larsen et al [27]. This concept aligns with European legal frameworks that prioritize glare reduction and reflection control over absolute darkness. Other disturbing factors, such as noise or non-ergonomic furniture, must also be addressed when designing an optimal workplace. Future studies should investigate how digital radiology technologies interact with ambient lighting to optimize both image interpretation and occupational health.

Daylight deprivation impacts interlinked physiological and psychological processes, including circadian regulation, mood, sleep, fatigue, and cognitive performance [8], all of which lead to potential malpractice [47]. Radiologists are particularly vulnerable to diagnostic errors based on their written reports, potentially triggering costly malpractice claims, a phenomenon described as Malpractice Stress Syndrome [48]. The combined effect of high cognitive load, low ambient light, and professional isolation significantly amplifies work-related stress, underscoring the urgent need for targeted interventions to protect mental health and maintain diagnostic quality in this high-risk group [49].

High burnout rates have been reported among radiologists [1], although some studies suggest rates similar to those of other medical specialties [50]. Radiologists, however, often report lower job satisfaction and perceive their work as less meaningful, partly due to interdisciplinary undervaluation [50]. This perception is reinforced by medical student surveys, which show many are deterred from radiology by its stereotypical image as “counting coins in the dark” [9], a factor exacerbating workforce shortages despite rising imaging demands [10]. Besides affecting the radiologists themselves, burnout causes costs through sick leave and early retirement [51, 52].

Radiologists are often stereotyped as providers of imaging reports, yet modern practice is far more multifaceted. Radiologists actively engage in clinical consultations (e.g., breast imaging), perform image-guided interventions, contribute to multidisciplinary boards, and play key roles in patient management, screening programs, and direct communication with clinicians and patients. As emphasized in the European Society of Radiology White Paper, the profession has evolved over recent decades, positioning radiologists as doctors, communicators, innovators, scientists, and teachers—shifting from isolated reporting to central contributors in therapeutic decision-making and patient care [53].

**Fig. 1:**
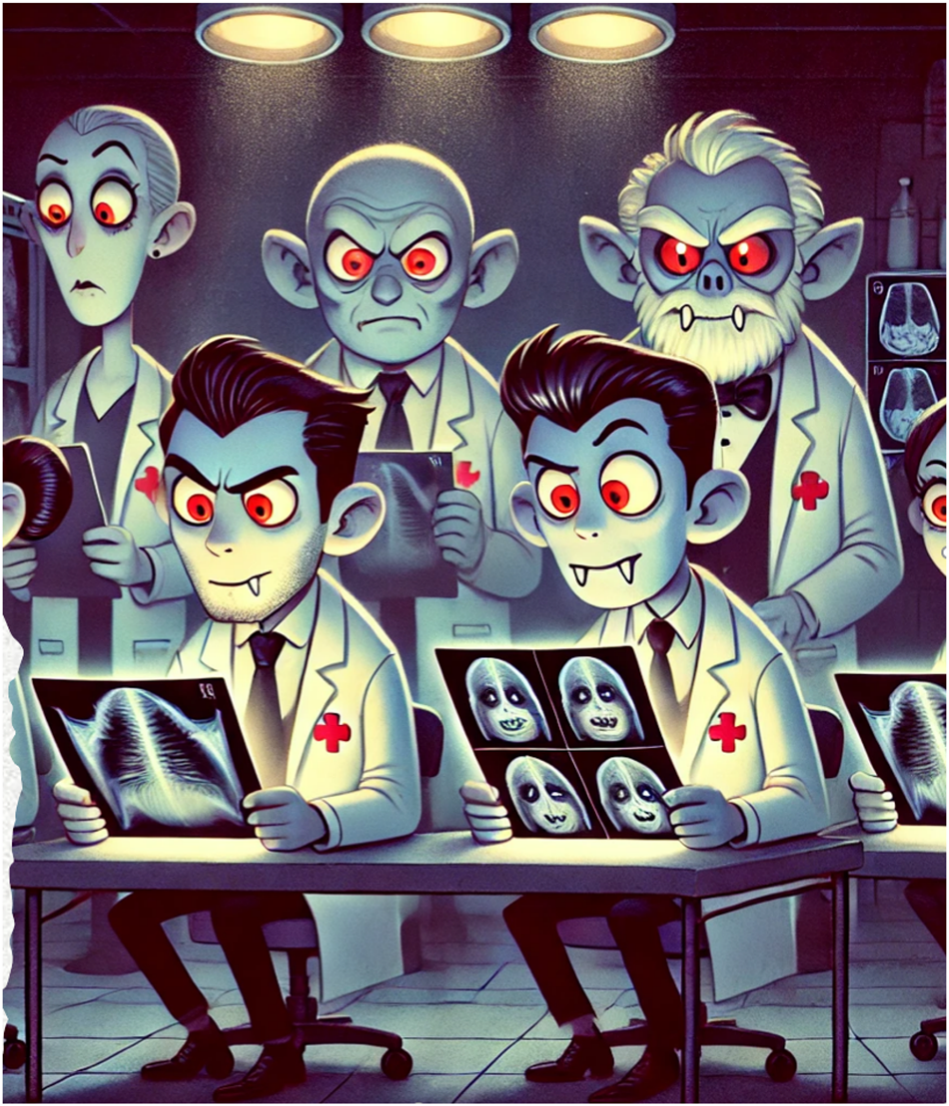
Artificial intelligence is often perceived as a threat to radiology, reinforcing fears of redundancy and discouraging new professionals from entering the field. The AI-generated image humorously exaggerates the stereotype of radiologists working in the dark, further distorting public perception. (Image was generated using DALL·E 3 (OpenAI) via the ChatGPT interface.)

In conclusion, the persistent stereotype of radiologists as isolated professionals working in dark rooms (as depicted in Fig. 1) has shaped architectural and organizational decisions, often prioritizing diagnostic needs over well-being despite evidence of health risks from limited daylight exposure. We recommend practical, low-cost improvements, such as adequate and adjustable ambient lighting or zoned collaborative areas with natural daylight during breaks (Table 2). These measures can reduce computer vision syndrome and support mood to enhance diagnostic accuracy and improve workforce sustainability [45, 54]. Future directions include longitudinal studies on intervention effects, pilot programs in European departments, and advocacy for updated EU guidelines that unify and better balance occupational health with modern digital imaging needs.

**Table 2:** Measures to improve the light environment in radiology departments

Measure	Description	Proposed benefits
Improved ambient lighting	Use dimmable neutral LED lights to maintain illumination of 20–40 lux; install blackout blinds for consistency.	Minimizes eye strain, visual fatigue, and improves diagnostic accuracy by matching monitor brightness (≥ 350 cd/m²).
Bias/back lighting	Position soft, indirect lights behind monitors and avoid overhead lighting to create wall glow without screen glare.	Stabilizes visual adaptation, reduces contrast mismatch, and promotes alertness in dim environments.
Individual workstation controls	Provide personal dimmers and task lights (e.g., desk lamps) for reading vs non-reading tasks.	Allows customization based on subspecialty (e.g., lower lux for mammography).
Collaboration areas	Incorporate zoned areas for breaks and other non-reading tasks with natural or simulated daylight.	Fosters collaboration and light exposure during non-diagnostic activities; enhances overall well-being and improves workforce sustainability; integrates with redesigns for multifaceted radiology roles.

## Abbreviations

cd/m^2^: Candela per square meter (unit of measurement for brightness)
DIN: Deutsches Institut für Normung (German Institute for Standardization)
LED: Light-emitted diode
SUVA: Schweizer Unfallversicherungsanstalt (Swiss National Accident Insurance Fund)
